# Single-cell mapping of tumor-driven macrophage reprogramming via ETV4-MC1R underlies immunotherapy resistance in colorectal cancer

**DOI:** 10.1097/JS9.0000000000004270

**Published:** 2026-02-25

**Authors:** Rui Liang, Yang Zhang, Lu Xing, Wenbin Wang, Yunfeng Li, Tao Shen, Xinyi Cai, Zhaoyu Yang, Jibiao Li, Xiaotao Yang, Xuan Zhang, Tao Wu

**Affiliations:** a Department of Colorectal Surgery, The Third Affiliated Hospital of Kunming Medical University, Yunnan Cancer Hospital, Peking University Cancer Hospital Yunnan, Kunming, China; b Department of Bioengineering, College of Bioengineering, Chongqing University, Key Laboratory of Biorheological Science and Technology, Ministry of Education, Chongqing, China; c Department of Vascular Surgery, Affiliated Cardiovascular Hospital of Kunming Medical University, Kunming, Yunnan, China; d Department of Dermatology, Children’s Hospital Affiliated to Kunming Medical University, Kunming, China; e Department of Public Health, School of Public Health, Kunming Medical University, Cheng Gong New City, Kunming, China

**Keywords:** colorectal cancer (CRC), ETV4, immunotherapy resistance, MC1R, tumor microenvironment (TME)

## Abstract

**Background:**

Immunotherapy resistance remains a major clinical challenge in advanced microsatellite instability-high (MSI-H) colorectal cancer (CRC), and the underlying gene regulatory networks (GRNs) distinguishing PD-1-resistant from PD-1-sensitive tumors are poorly defined.

**Methods:**

We performed single-cell RNA sequencing on tumor samples from three PD-1-resistant and three PD-1-sensitive MSI-H metastatic CRC patients. Resistance-associated transcription factors were identified by integrating machine learning-based prognostic modeling with GRN reconstruction. Bioinformatic findings were functionally validated using dual-luciferase reporter assays, chromatin immunoprecipitation, and *in vitro* co-culture experiments. Furthermore, the therapeutic efficacy of co-targeting the ETV4-MC1R axis with anti-PD-1 treatment was evaluated *in vivo*.

**Results:**

Several transcriptional regulators were highly active in resistant tumors, among which the transcription factor ETV4 was markedly upregulated in malignant epithelial cells and played a central role. A prognostic model based on ETV4 downstream target genes effectively stratified CRC into subtypes with distinct outcomes. Transcriptome-wide correlation analysis revealed a strong positive association between ETV4 expression and macrophage markers. Mechanistically, we identified MC1R as a critical downstream target of ETV4 mediating resistance. Functional assays confirmed direct binding and activation of the MC1R promoter by ETV4. Co-culture experiments demonstrated that ETV4-high CRC cells promoted macrophage polarization toward an immunosuppressive M2 phenotype via the MC1R pathway. *In vivo* validation showed that combining ETV4-MC1R targeting with anti-PD-1 therapy significantly reduced tumor volume by 55.6% compared to anti-PD-1 monotherapy (*P* < 0.05).

**Conclusion:**

This study reconstructs the regulatory network underlying PD-1 resistance in CRC at single-cell resolution and reveals a novel tumor-intrinsic ETV4-MC1R signaling axis that drives M2 macrophage polarization and immune evasion. Targeting this pathway presents a promising combination strategy to overcome immunotherapy resistance in CRC.

## Introduction

Colorectal cancer (CRC) remains a major global health burden, ranking as the third most prevalent malignancy and second leading cause of cancer-related mortality worldwide^[1]^. Although advancements in screening and therapeutic interventions have been achieved, the 5-year overall survival rate for patients with CRC remains unsatisfactory^[2]^. Notably, patients with metastatic CRC have substantially lower 5-year survival rates than those diagnosed at earlier stages^[3]^. Single-cell analyses have revealed significant differences in the tumor microenvironment (TME) between CRC and normal tissues, with marked intratumoral heterogeneity observed across different disease stages. This dynamic TME critically influences tumorigenesis, progression, metastatic dissemination, and therapeutic resistance^[4–7]^. Recent single-cell studies have highlighted the role of specific factors in TMEs; for instance, ILF2 was identified as a key regulator in breast cancer brain metastasis^[8]^, while CD20⁺ B cells were shown to enhance anti-PD-1 efficacy in CRC^[9]^.

Recent breakthroughs in immunotherapy have shown promise for the management of CRC. Mismatch repair deficiency (dMMR) and high microsatellite instability (MSI-H) status have emerged as predictive biomarkers for the efficacy of immune checkpoint blockade, with improved objective response rates (ORRs) observed in patients with MSI-H metastatic CRC (mCRC)^[10,11]^. However, first-line anti-programmed cell death protein 1 (PD-1) monotherapy achieves only 43.8% ORR in MSI-H/dMMR mCRC patients^[12]^, underscoring the critical need to elucidate the mechanisms underlying PD-1 resistance in this population.

Cellular transcriptional states are governed by gene regulatory networks (GRNs) comprising transcription factors (TFs), co-regulators, and their target genes. The advent of single-cell RNA sequencing (scRNA-seq) has enabled GRN reconstruction through regulatory network inference and TF clustering, thereby providing unprecedented insights into dynamic cellular state transitions during tumor evolution^[13]^. Emerging studies employing scRNA-seq-based GRN analyses have identified distinct transcriptional programs in tumor cell populations, providing novel insights into oncogenic mechanisms^[14]^. Nevertheless, the dynamics of GRN in PD-1-resistant CRC remains unclear, necessitating further investigation and experimental validation.

**Figure 1.**
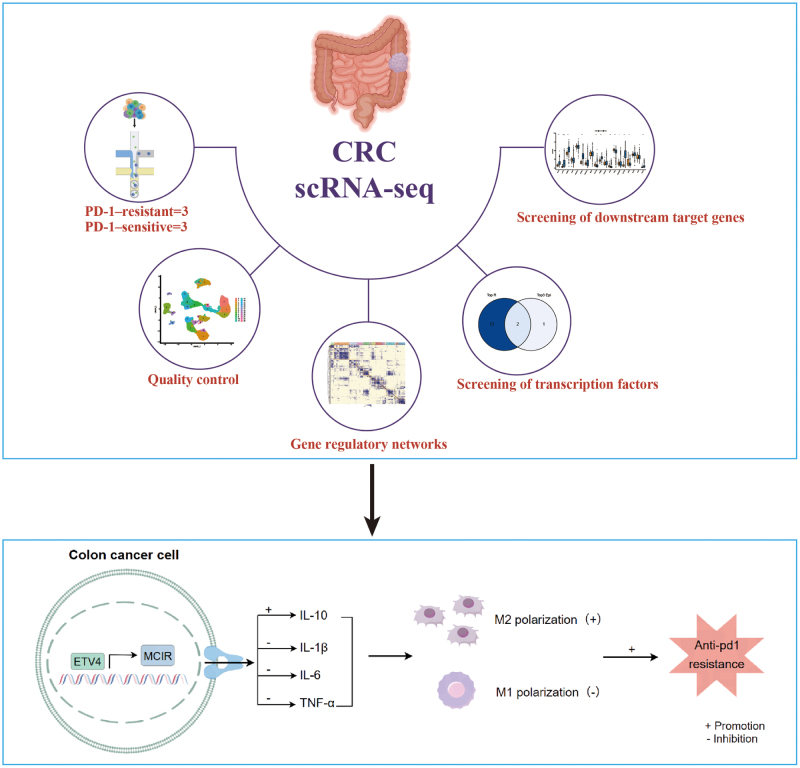
The study design and analysis workflow diagram.

This study integrated scRNA-seq data from PD-1 blocker-treated MSI-H mCRC patients (including sensitive and resistant patients) for systematic GRN analysis. We identified resistance-specific TFs with increased transcriptional activity, particularly ETV4, which is a key regulator expressed primarily in malignant epithelial cells. TCGA dataset analysis revealed potential associations between ETV4 expression and macrophage-related signatures, prompting subsequent investigations of the role of ETV4 in macrophage polarization in co-culture systems. These findings were corroborated by murine models. Mechanistically, we demonstrated that ETV4 confers PD-1 resistance in CRC via transcriptional activation of MC1R, which was validated by comprehensive *in vitro* and *in vivo* experiments. Our results indicate that the ETV4-MC1R axis is a crucial mediator of immune evasion and is a promising therapeutic target for overcoming PD-1 resistance in CRC. Our study design and analytical workflow are summarized in Figure 1. We declare that we have not used any artificial intelligence in our research and manuscript development and comply with TITAN Guidelines 2025^[15]^.

## Methods

### Data preparation and cleaning

scRNA-seq data were generated in-house from tumor specimens of three PD-1-resistant and three PD-1-responsive CRC patients and were deposited in the Gene Expression Omnibus (GEO)^[4]^. In addition, bulk CRC transcriptomic datasets with accompanying survival information (GSE17536, GSE17537, and GSE38832) were downloaded from GEO for downstream analyses^[16–18]^. During preprocessing, cells with <200 detected genes, >7000 detected genes, or >5% ribosomal RNA reads were discarded. The 2000 most highly variable genes were retained for downstream analysis. Highly variable genes were identified with the “vst” method from the Seurat package using the FindVariableFeatures function. This approach fits a curve to the relationship between log(variance) and log(mean) through local polynomial regression (loess), standardizes gene expression values using the observed mean and expected variance from the fitted model, and then calculates feature variance on the standardized values after applying a clip threshold (as set by the clip.max parameter). Markers used for cell-type annotation are provided in Supplemental Digital Content Figure 1A, available at: http://links.lww.com/JS9/G974, and cell proportion plots are shown in Supplemental Digital Content Figure 2C, available at: http://links.lww.com/JS9/G974. Batch effects and interpatient variability were corrected using the Harmony R package. The cell types were annotated manually using established marker genes from the literature and CellMarker database^[19]^. All visualizations were created using the R package SCP.

**HIGHLIGHTS**

- Single-cell transcriptomics identified ETV4 as a key driver of PD-1 resistance in high microsatellite instability colorectal cancer.
- ETV4 activity is enriched in malignant epithelial cells and linked to macrophage signatures.
- ETV4 promotes immune evasion by skewing macrophages to an M2 phenotype via MC1R.
- Targeting the ETV4–MC1R axis restores antitumor immunity and inhibits tumor growth.
- The ETV4–MC1R pathway is a promising target for overcoming immunotherapy resistance.

### TF gene regulation analysis and visualization

Single-cell regulatory network inference and clustering (SCENIC) was used to reconstruct GRNs and define cell states from single-cell transcriptomic data by integrating gene co-expression with cis-regulatory motif analysis^[20]^. The curated list of human TFs was downloaded from the cisTarget repository (https://resources.aertslab.org/cistarget). Gene-regulatory network inference was carried out using pySCENIC v0.9.18, and motif enrichment (cisTarget) analysis was carried out using pySCENIC v0.12.1. Initially, putative TF-target interactions were inferred by constructing gene co-expression networks, which served as the basis for subsequent regulatory motif enrichment and network refinement.

### Constructing a prognostic model via multiple machine learning

The feature set used to construct the prognostic model comprised the intersection of CRC-related genes identified by univariate Cox analysis and downstream target genes of ETV4. In total, nine machine learning algorithms were applied across 101 model combinations^[21,22]^. The integrated algorithms included random survival forest (RSF), elastic net (ENet), lasso, ridge, stepwise Cox regression, CoxBoost, partial least-squares Cox regression (PLS-RCox), supervised principal component analysis (SuperPC), gradient boosting machine, and survival support vector machine. Kaplan-Meier curves were plotted to compare the overall survival between the high- and low-risk patient groups.

### Biological differences between high-risk and low-risk patients

We assessed biological differences between the high- and low-risk groups. For stratified cohorts, single-sample gene set enrichment analysis (ssGSEA) was used to compare immune cell infiltration profiles and clinical characteristics. Using ssGSEA, we evaluated the scores of 13 programmed cell death pathways and 13 immune cell signatures in the high-risk group^[23,24]^. ssGSEA was employed to investigate the relationships between immune cell infiltration, clinical characteristics, and ETV4 expression in patients stratified into high- and low-expression groups.

### Animal experiments

All animal procedures were approved by the Animal Ethics Committee of Kunming Medical Uni and conducted in strict compliance with the ARRIVE guidelines^[25]^. Investigators were blinded to group assignments throughout animal allocation, experimental procedures, and outcome assessment.

Male BALB/c mice (6–8 weeks old, 20–30 g) were obtained from Beijing Sipeifu Biotechnology Co., Ltd., and housed under specific pathogen-free conditions at Kunming Medical University with ad libitum access to food and water. After a 3-day acclimatization period, experiments were initiated.

**Sample size was determined using Mead’s degrees of freedom method for variance analysis, with power ≥80% and α = 0.05. Based on preliminary data and expected effects, a total sample size of 42 mice (6 per group) was deemed sufficient to detect significant intergroup differences while adhering to the 3Rs principles.** Mice were randomly assigned to seven experimental groups via simple random sampling: (1) NC group: empty vector control cells; (2) sh-MC1R group: MC1R-knockdown cells; (3) NC + RMP1-14 group: control cells + anti-PD-1 antibody; (4) OE-ETV4 group: ETV4-overexpressing cells; (5). OE-ETV4 + RMP1-14/nivolumab group: ETV4-overexpressing cells + anti-PD-1 antibody; (6) OE-ETV4 + sh-MC1R + RMP1-14/nivolumab group: ETV4-overexpressing + MC1R-knockdown cells + anti-PD-1 antibody; **7. OE-ETV4 + PLX3397 group: ETV4-overexpressing cells + CSF1R inhibitor (depletes TAMs).**

Each mouse received a subcutaneous injection of 1 × 10^6^ respective stable transfected cells in 100 µL PBS into the right flank. Anti–PD-1 treatment began when tumor volumes reached approximately 40 mm^3^ (day 11 post-inoculation). The NC + RMP1-14, OE-ETV4 + RMP1-14/nivolumab, and OE-ETV4 + sh-MC1R + RMP1-14/nivolumab groups were administered RMP1-14 or nivolumab via intraperitoneal injection twice weekly. Control groups received PBS following the same schedule. To evaluate TAM dependency, the OE-ETV4 + PLX3397 group received PLX3397. To evaluate TAM dependency, the OE-ETV4 + PLX3397 group received PLX3397 (Med Chem Express, 50 mg/kg, qod) via oral gavage starting on day 11. PLX3397 is a selective CSF1R inhibitor that depletes macrophages by blocking CSF1R-mediated survival signaling.

Tumor dimensions and mouse body weights were measured at days 0, 7, 10, 13, 16, 19, 22, 25, 28, and 32. Tumor volume was calculated using the formula: V = ½ × (Length × Width²). The experiment was terminated on day 32 post-inoculation, based on pre-experimental optimization. Throughout the study, tumor length did not exceed 20 mm, and tumor weight remained below 10% of total body weight. All mice were euthanized under anesthesia, and tumor tissues were collected for subsequent molecular analyses.

### Cell co-culture

Cell co-culture was performed using transwell chambers. THP-1 cells in the logarithmic growth phase were adjusted to a density of 1 × 10^5^ cells/mL and seeded into the upper chambers of a 12-well transwell plate. Phorbol myristate acetate PMA (final concentration: 185 ng/mL) was added to induce differentiation into M0 macrophages after 6 h of incubation. Lower chambers were loaded with 1 × 10 × HCT116/SW480 cells per well. Cells were co-cultured for 48 h before collection.

### RT-qPCR

Total RNA was isolated using TRIzol Reagent according to the manufacturer’s protocol. The quantity and purity of RNA were assessed using a NanoDrop ND-1000 spectrophotometer. Reverse transcription was performed using Hifair^®^ III 1st Strand cDNA Synthesis SuperMix for qPCR (YEASEN) under the following conditions: 42°C for 2 min, 25°C for 5 min, 55°C for 15 min, 85°C for 5 min, and hold at 4°C. The diluted cDNA (1:5) was amplified using the Hieff UNICON^®^ Universal Blue qPCR SYBR Green Master Mix (YEASEN) with the following cycling parameters: initial denaturation at 95°C for 2 min; 40 cycles of 95°C for 10 s (denaturation) and 60°C for 30 s (annealing/extension). Fluorescence signals were acquired during the extension step and relative gene expression was calculated via the 2^−ΔΔCt^ method. The primers used in this paper are detailed in Table 1.

**Table 1:** Sequences of the primers.

Gene	Forward (5′-3′)	Reverse (5′-3′)
ETV4	TCACACTCCCTCTGGGTGAA	GGTGACATCTGTGGGGGAAG
MC1R	GAGTAGCACCTGGGGTGAAC	AGGGCATCTCACTCCAGACT
TNF-α	ACCCTCACACTCACAAACCA	ATAGCAAATCGGCTGACGGT
IL-6	CCCCAATTTCCAATGCTCTCC	CGCACTAGGTTTGCCGAGTA
IL-1β	TGCCACCTTTTGACAGTGATG	AAGGTCCACGGGAAAGACAC
IL-10	TTGCATACGGGACAGAACTGC	CCAGGGTGAACGTTGTGAGA
GAPDH	CCTTCCGTGTTCCTACCCC	GCCCAAGATGCCCTTCAGT

### Western blot

The tissue samples were lysed in 500 μl of RIPA buffer (Servicebio, G2002) containing protease inhibitors, and the protein concentration was quantified using a BCA protein assay kit (Beyotime, P0009). Proteins were separated by 10% SDS-PAGE and transferred onto PVDF membranes. After blocking with 5% bovine serum albumin (BSA), the membranes were incubated overnight at 4°C with the following primary antibodies: CD86 (Affinity, DF6332, 1:2000), CD206 (Proteintech, 18 704-1-AP, 1:1000), ETV4 (Santa Cruz Biotechnology, sc-166 629, 1:1000), MC1R (Abcam, ab236734, 1:1000), and β-actin (Proteintech, 66 009-1-Ig, 1:25 000). Membranes were subsequently incubated with the following HRP-conjugated secondary antibodies: goat anti-rabbit IgG (Servicebio, GB23303, 1:3000) and goat anti-mouse IgG (Servicebio, GB23301, 1:5000). The protein bands were visualized using a high-sensitivity enhanced chemiluminescence kit and imaged using a Vilber BioImaging System. Band intensities were quantified using dedicated analysis software.

### Immunofluorescence

Tissues were fixed in 4% paraformaldehyde (Guangfu, CNAB035-Q) overnight, dehydrated using a graded ethanol series, cleared with xylene (Xilong Chemical Co., Ltd., 33 535), and embedded in paraffin. Sections (5 μm) were heated at 64°C (Shaoxing Yihui Instrument Co., Ltd., 303-2) for 1 h, deparaffinized in xylene, rehydrated with ethanol, and subjected to antigen retrieval using a citrate buffer (Servicebio, G0001-1 L). Endogenous peroxidase activity was quenched by incubation with permeabilization solution at room temperature for 20 min, followed by blocking with 5% BSA (Gibco) for 30 min. Primary antibodies against ETV4 (Santa Cruz Biotechnology, sc-166 629, 1:100), MC1R (Affinity, DF4992, 1:100), CD206 (Proteintech, 18 704-1-AP, 1:200), and CD86 (Affinity, DF6332, 1:100), diluted in 2% BSA were applied at 4°C overnight. The sections were then incubated with Alexa Fluor^®^-conjugated secondary antibodies (37°C for 20 min). Nuclei were stained with DAPI (Ex: 330–380 nm, Em: 420 nm; blue), ETV4 was labeled with Alexa Fluor^®^ 488 (Ex: 495 nm, Em: 519 nm; green), and MC1R, CD86, and CD206 were labeled with Alexa Fluor^®^ 555 (Ex: 555 nm, Em: 565 nm; red).

### Flow cytometry

The cells were collected and centrifuged at 300 × *g* for 5 min, after which the supernatant was discarded. After washing once with PBS (300 × *g* centrifugation for 5 min), the cells were resuspended at a density of 1 × 10^5^ cells per tube. Each sample tube was incubated with 100 μl of 1× binding buffer and stained with specific antibodies, including PE-conjugated anti-mouse CD86 (Elabscience, E-AB-F0994D), PE-conjugated anti-mouse CD206 (Elabscience, E-AB-F1135D), FITC-conjugated anti-human CD11b (BioLegend, 301 403), PE-conjugated anti-human CD86 (BioLegend, 374 205), and PE-conjugated anti-human CD206 (BioLegend, 321 105) in the dark at room temperature for 15 min. After 400 μl of 1× binding buffer was added and filtered through a cell strainer, the samples were analyzed using a BD FACSCalibur flow cytometer (10 000 events per sample). The data were processed using FlowJo software to quantify the protein expression levels.

### ChIP-PCR

Chromatin immunoprecipitation (ChIP) was performed using an ETV4-specific antibody according to the ChIP kit protocol (Active Motif, 53 009). The precipitated DNA was purified, and the promoter sequence of the MC1R gene was identified using the UCSC Genome Browser (https://www.genome.ucsc.edu/). The putative ETV4 binding sites were predicted using the JASPAR database (https://jaspar.elixir.no/). Specific primers flanking the predicted binding regions were designed for the PCR amplification of ChIP-enriched DNA. The amplification products were analyzed by agarose gel electrophoresis and validated by Sanger sequencing to assess binding site enrichment.

### Dual-luciferase reporter assay

MC1R wild type (WT) and mutant (MUT) dual-luciferase reporter plasmids were synthesized by Auno Biological. 293 T cells cultured in 96-well plates (70%–80% confluency) were transfected with plasmids for 48 h. After washing with PBS, cells were lysed with 20 μl of 1× lysis buffer per well. Luciferase activity was measured using a Dual-Luciferase Reporter Assay Kit (Yeasen, 11402ES60) according to the manufacturer’s protocol. Firefly luciferase activity was normalized to the Renilla luciferase activity for quantitative analysis.

### ELISA

All reagents, samples, and standards were processed according to the ELISA kit protocol for the detection of target genes. Each well was loaded with 50 μl of the diluted sample, followed by the immediate addition of 100 μl of the prepared enzyme-conjugated reagent A. After thorough mixing, plates were incubated at 37°C for 1 h. After incubation, the wells were aspirated and washed five times. Subsequently, 100 μl of the prepared detection reagent was added to each well, and the plate was incubated at 37°C for 30 min. Following aspiration and five washing cycles, 50 μl of chromogenic substrate A and 50 μl of chromogenic substrate B were sequentially added. The plates were incubated in the dark at 37°C for 15 min to facilitate color development. The reaction was terminated with 50 μl of stop solution, and the absorbance was measured at 450 nm using an ELx800 microplate reader.

### Statistical analysis

Bioinformatics analyses were performed using R software. Data analysis was performed using GraphPad Prism software, and the experimental results were expressed as the mean ± standard error of the mean (mean ± SEMs). Two-tailed one-way analysis of variance with multiple comparison post hoc analysis was used, and *P* < 0.05 (*), P <  0.01 (**), P < 0.001 (***), and P < 0.0001 (****) are indicated as significant.

## Results

### GRNs in PD-1-resistant and PD-1-sensitive CRC

**Figure 2.**
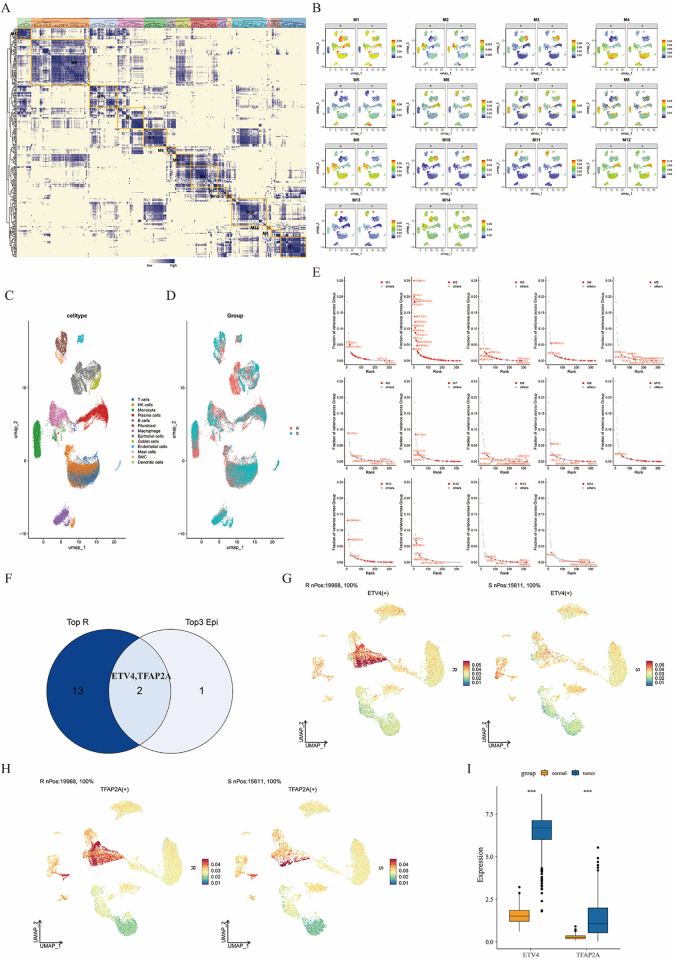
Distinct transcriptional regulatory landscapes underlying PD-1 resistance and sensitivity in colorectal cancer (CRC). (A) The heatmap displays transcription factor (TF) clustering to identify modules with high transcriptional activity. (B) Uniform manifold approximation and projection (UMAP) showing the distribution of transcription factor modules in PD-1-resistant and PD-1-sensitive CRC patients. (C and D) UMAP visualizes the distribution of CRC gene subgroups and clusters. (E) Transcription factor expression across different modules. (F) Venn diagram illustrating the overlap between transcription factors with high expression and high transcriptional activity in epithelial cells. (G and H) UMAP plots of ETV4 and TFAP2A expression in PD-1-resistant and PD-1-sensitive CRC. (I) Boxplot comparing ETV4 and TFAP2A expression in adjacent normal tissues and colorectal cancer tissues in The Cancer Genome Atlas (TCGA) cohort. UMAP, Uniform Manifold Approximation and Projection; TF, transcription factor; TCGA, The Cancer Genome Atlas; CRC, colorectal cancer.

First, we performed quality control on scRNA-seq data. A heatmap illustrates the expression of marker genes used for cell type annotation (Supplemental Digital Content Figure 1A, available at: http://links.lww.com/JS9/G974), and UMAP plots reveal the distributions of various cell populations (Supplemental Digital Content Figures 1B-C, available at: http://links.lww.com/JS9/G974). We then analyzed the transcriptional regulatory differences between the PD-1-resistant and PD-1-sensitive CRC samples. A heat map revealed 14 distinct transcriptional modules (Fig. 2A). Among them, UMAP visualization indicated that Module M1 presented the most significant differences in TF activity between the PD-1-resistant and PD-1-sensitive groups (Fig. 2B). The UMAP plots also demonstrated the distribution of cellular subpopulations across PD-1-resistant and PD-1-sensitive CRC samples (Fig. 2C-D). Next, we examined the TFs with the highest activity across the individual modules (Fig. 2E). Among these TFs, both ETV4 and TFAP2A exhibited markedly elevated activity in epithelial cells in the PD-1-resistant CRC group (Fig. 2F-H). Analysis of TCGA transcriptome data further revealed that ETV4 expression in CRC tissues was significantly greater than TFAP2A expression in the control group (Fig. 2I). Using ssGSEA, we assessed ETV4 transcriptional activity and evaluated its prognostic relevance in the IMvigor210 immunotherapy cohort. Our analysis demonstrated that elevated ETV4 activity was significantly associated with worse clinical outcomes across the entire cohort, with this association being particularly pronounced in the immunotherapy-resistant subgroup (Supplemental Digital Content Figures 2A-B, available at: http://links.lww.com/JS9/G974). These findings suggest that the TF ETV4 may play a critical role in mediating PD-1 resistance in CRC.

### Construction of a prognostic model based on downstream target genes of the TF ETV4

**Figure 3.**
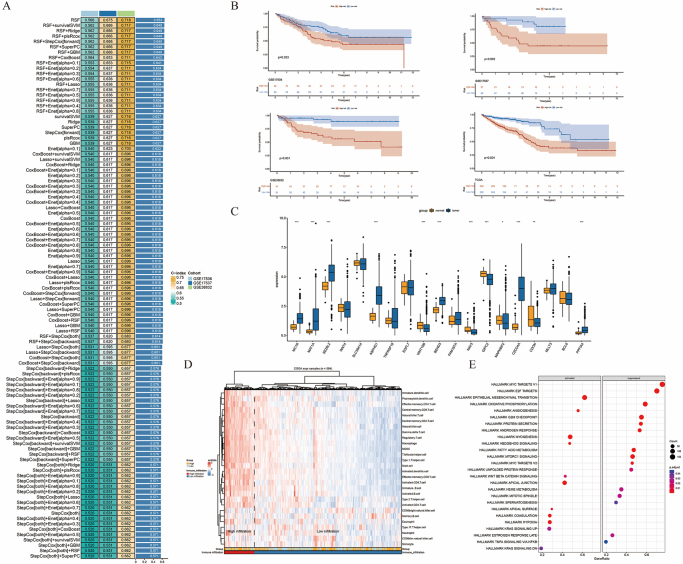
Prognostic model construction based on ETV4-regulated downstream target genes. (A) The heatmap displays the optimal prognostic models identified through various machine learning methods. (B) Kaplan-Meier (KM) survival analysis showing the prognosis of high-risk groups of CRC patients. (C). Boxplot comparing the expression levels of model genes between adjacent normal tissues and colorectal cancer tissues in TCGA. (D) Heatmap illustrating the relationships among high-risk/low-risk groups, clinical features, and immune cell infiltration. (E) Bar plot depicting the pathway enrichment analysis of DEGs in the high-risk group versus the low-risk group using gene set enrichment analysis (GSEA). RSF, random survival forest; C-index, concordance index; GSEA, gene set enrichment analysis.

We employed multiple machine learning algorithms to construct a prognostic model based on the downstream target genes of ETV4, using three independent clinical cohorts. Among the 101 combinations derived from nine machine learning algorithms, the RSF model demonstrated the best performance, achieving an average concordance index (C-index) of 0.653 across both the training and validation cohorts (Fig. 3A). Moreover, the prognostic analysis across several clinical cohorts consistently revealed that patients with high-risk scores had significantly poorer outcomes (Fig. 3B). Further analysis of gene expression in TCGA cohort revealed that most of the genes included in the RSF model were significantly upregulated in CRC samples (Fig. 3C). We then performed an immune cell infiltration analysis based on the constructed ETV4 downstream target gene-derived prognostic model. The heatmap revealed that patients with high-risk scores had an increased infiltration of immune cells (Fig. 3D), consistent with these findings, immune cell deconvolution analysis revealed significantly higher macrophage-related scores in the high-risk group (Supplemental Digital Content Figure 1D, available at: http://links.lww.com/JS9/G974). Additionally, gene set enrichment analysis revealed that multiple tumor progression-related pathways were significantly enriched in the high-risk subgroup (Fig. 3E). These findings suggest that genes defining the high-risk group play important roles in CRC progression and may be associated with macrophage polarization.

### ETV4 is associated with PD-L1 expression and macrophage recruitment

**Figure 4.**
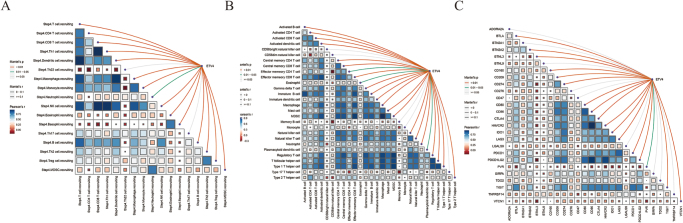
ETV4 correlates with PD-L1 expression and macrophage infiltration. (A and B) The heatmap shows the relationship between ETV4 and TIP-related immune cell infiltration analyzed by single-sample gene set enrichment analysis (ssGSEA). (C) The heatmap shows the relationships between ETV4 and immune checkpoints including programmed cell death 1 (PDCD1) and PDCD1 ligand 2 (PDCD1LG2). TIP, tumor immunophenotype; ssGSEA, single-sample gene set enrichment analysis; PDCD1, programmed cell death 1; PDCD1LG2, PDCD1 ligand.

Using TCGA datasets, we evaluated the relationship between ETV4 expression and immune checkpoint gene expression. ETV4 was positively correlated with several checkpoints, most notably PDCD1 and PDCD1LG2 (Fig. 4A). These findings are consistent with our single-cell screening results, underscoring the potential importance of ETV4 in PD-1 resistance in CRC.

Subsequent ssGSEA of TCGA CRC samples revealed a significant positive association between ETV4 expression and macrophage signatures (Fig. 4B). Furthermore, TIP analysis confirmed that ETV4 expression was strongly correlated with macrophage recruitment in CRC (Fig. 4C). Collectively, these results suggest that ETV4 contributes to PD-1 resistance in CRC by modulating macrophage recruitment.

### ETV4 transcriptionally activates MC1R through direct promoter binding

**Figure 5.**
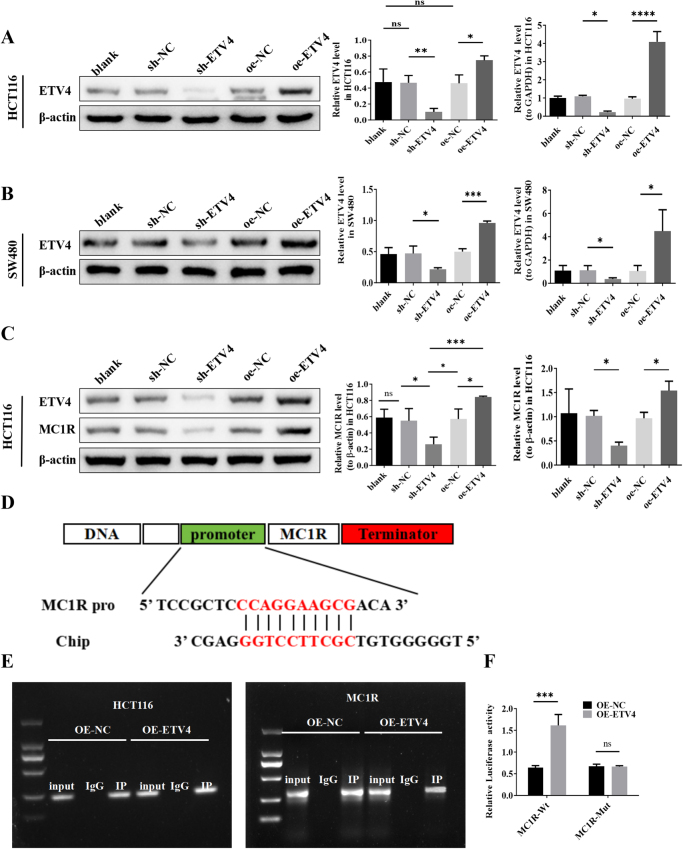
ETV4 transcriptionally activates MC1R via direct binding to its promoter region. (A) QPCR and western blot validation of ETV4 knockdown (shETV4) and overexpression (oe-ETV4) in HCT116 cells. (B) QPCR and western blot validation of ETV4 knockdown (shETV4) and overexpression (oe-ETV4) in SW480 cells. (C) QPCR and western blot analysis of MC1R expression upon ETV4 modulation. (D) Bioinformatic prediction of ETV4-binding motifs in the MC1R promoter via University of California Santa Cruz (UCSC) and JASPAR. (E) ChIP assay demonstrating that ETV4 binds to the MC1R promoter. (F) Dual-luciferase reporter assay assessing ETV4-mediated MC1R promoter activity. ChIP, chromatin immunoprecipitation; UCSC, University of California Santa Cruz.

Successful ETV4 knockdown (shETV4) and overexpression (oe-ETV4) in HCT116 and SW480 cells were confirmed (Fig. 5A-B). Seven downstream target genes of ETV4 that were significantly differentially expressed in cancerous tissues are shown in Figure 3C: MC1R, MAT1A, SEZ6L2, ASPHD1, BEND3, C2CD4A, and PPFIA4. Among these candidates, MC1R was selected for further investigation due to its potential relevance to tumor–immune interactions. Therefore, we investigated whether ETV4 promoted PD-1 resistance in CRC through the MC1R pathway. Functional studies in CRC cells have revealed that ETV4 regulates MC1R expression. ETV4 overexpression significantly increased MC1R levels, whereas ETV4 silencing suppressed MC1R expression (Fig. 5C).

To investigate the mechanism underlying this regulation, bioinformatic analysis was used to predict potential ETV4-binding motifs in the MC1R promoter region using the UCSC genome browser and JASPAR databases (Fig. 5D). ChIP assays demonstrated that ETV4 binds to the endogenous MC1R promoter (Fig. 5E). Dual-luciferase reporter assays revealed that ETV4 overexpression enhanced the activity of the wild-type MC1R promoter compared to that of the controls. Mutation of the predicted ETV4-binding site abolished this activation (Fig. 5F).

To functionally validate MC1R pathway activation, we performed loss-of-function experiments in CT26 cells. MC1R knockdown significantly altered macrophage polarization with decreased CD206⁺ M2 populations and increased CD86⁺ M1 populations (Supplemental Digital Content Figure 3, available at: http://links.lww.com/JS9/G974). Concurrently, cytokine analysis revealed elevated pro-inflammatory cytokines (IL-1β, IL-6, and TNF-α) and reduced anti-inflammatory IL-10 compared to sh-NC controls (Supplemental Digital Content Figure 4A, available at: http://links.lww.com/JS9/G974). These findings establish that ETV4 is a direct transcriptional activator of MC1R and demonstrate functional activation of the MC1R signaling pathway.

### ETV4 drives M2 macrophage polarization to promote immunosuppression

**Figure 6.**
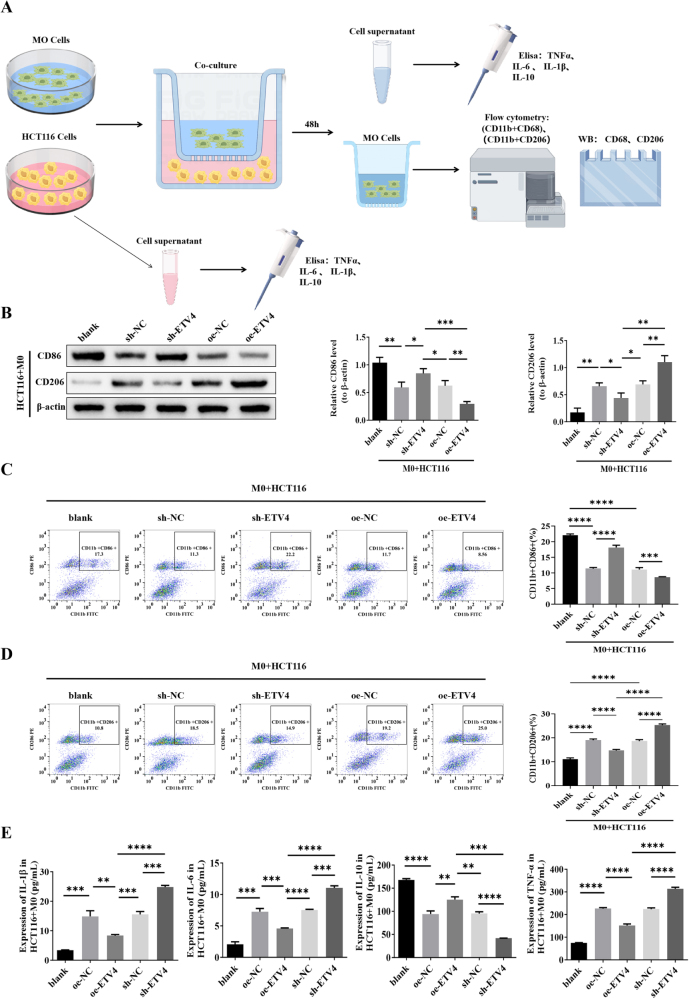
ETV4 drives M2 macrophage polarization to facilitate an immunosuppressive tumor microenvironment. (A) Schematic of the co-culture system (HCT116 cells ± ETV4 modulation with M0 macrophages). (B) Western blot analysis of M1 (CD86) and M2 (CD206) macrophage markers. (C and D) Flow cytometry quantification of CD11b⁺CD86⁺ (M1) and CD11b⁺CD206⁺ (M2) macrophages. (E) Enzyme-linked immunosorbent assay (ELISA) measurement of cytokine secretion (IL-1β, IL-6, TNF-α, and IL-10) in co-cultured macrophage supernatants.

These findings suggest that ETV4 modulates PD-1 resistance in CRC via its effects on macrophages. To investigate the role of ETV4 in macrophage polarization, a co-culture system was established using HCT116 cells (ETV4 knockdown or overexpression) and M0 macrophages (Fig. 6A).

Macrophages co-cultured with ETV4-silenced HCT116 cells showed upregulated M1 markers (CD86) and downregulated M2 markers (CD206), whereas those co-cultured with ETV4-overexpressing HCT116 cells showed suppressed CD86 and elevated CD206 expression (Fig. 6B). Flow cytometry confirmed these polarization shifts: ETV4 knockdown increased the proportion of CD11b⁺CD86⁺ M1-like macrophages and reduced the number of CD11b + CD206⁺ M2-like cells, whereas ETV4 overexpression reversed this trend (Fig. 6C-D).

Functional validation via ELISA further demonstrated that ETV4 knockdown enhanced the secretion of M1-associated cytokines (IL-1β, IL-6, and TNF-α) and suppressed M2-associated IL-10 in macrophage supernatants, with ETV4 overexpression producing an opposite cytokine profile (Fig. 6E). Notably, the cytokine expression trends in HCT116-conditioned media directly mirrored those in co-cultured macrophage supernatants (Supplemental Digital Content Figure 4B, available at: http://links.lww.com/JS9/G974), confirming that ETV4-expressing CRC cells drive macrophage reprogramming toward an immunosuppressive M2 phenotype.

### The ETV4-MC1R axis drives tumor progression and modulates response to PD-1 therapy in CRC

**Figure 7.**
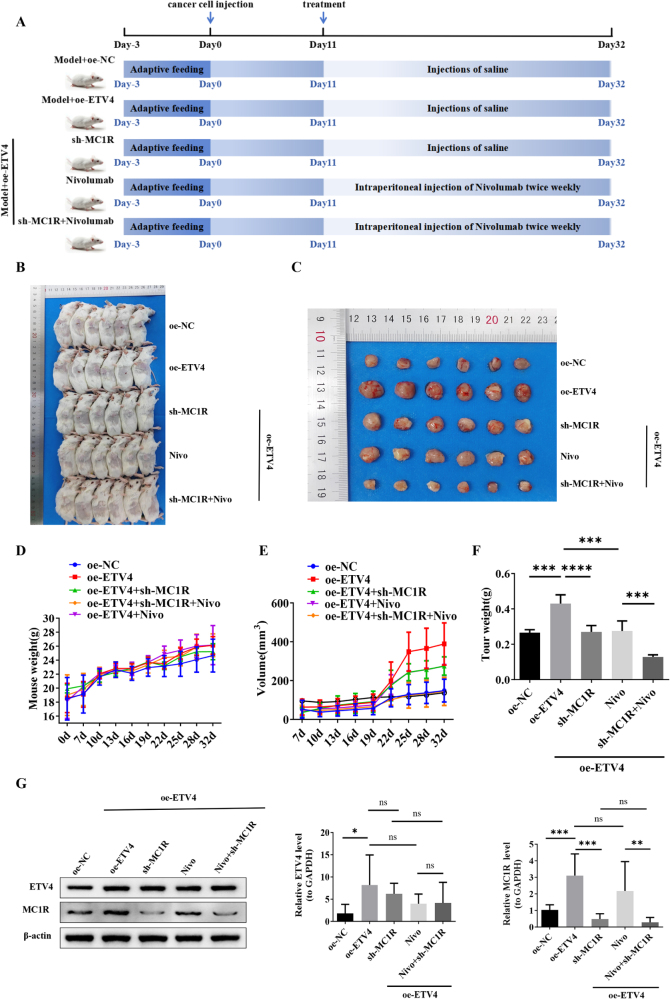
The ETV4–MC1R axis promotes tumor progression and shapes PD-1 therapy response in colorectal cancer. (A) Diagram of the experimental timeline: CT26 xenograft model with different interventions. (B and C) Xenograft tumors of BALB/c mice 32 days after inoculation with CT26 cells containing an empty vector or ETV4 overexpression vector (*n* = 6). (D–E) Average body weight and average tumor volume of BALB/c mice in each treatment group (*n* = 6). (F) Tumor weight across treatment groups (*n* = 6). (G) qPCR and western blot analysis of ETV4/MC1R expression in tumors (*n* = 6).

To investigate the roles of ETV4 and MC1R in CRC progression, we established a comprehensive *in vivo* xenograft model. Forty-two male BALB/c mice were randomly divided into seven groups (six mice per group) and subcutaneously implanted with CT26 cell lines with various genetic modifications: control (NC), MC1R knockdown (sh-MC1R), ETV4-overexpressing (oe-ETV4), or ETV4-overexpressing with MC1R knockdown (oe-ETV4 + shMC1R). Anti-PD-1 therapy (nivolumab or RMP1-14, twice weekly) and PLX3397 (administered by oral gavage for TAM depletion) were initiated on day 11 post-inoculation for designated groups, with tumors harvested on day 32 (Fig. 7A and Supplemental Digital Content Figure 5A, available at: http://links.lww.com/JS9/G974).

Longitudinal measurements of tumor volume at multiple time points (7 d, 10 d, 13 d, 16 d, 19 d, 22 d, 25 d, 28 d, and 32 d) are presented in Figure 7E. The results demonstrate accelerated tumor growth in the ETV4-overexpressing group, while the combination treatment group showed significantly suppressed tumor growth beginning at day 19.

As shown in Figure 7B-F, ETV4 overexpression significantly increased tumor volume and weight. No significant differences in mouse body weight were observed among the groups. Supplemental Digital Content Figures 5B-F, available at: http://links.lww.com/JS9/G974 show that the sh-MC1R monotherapy group exhibited a tumor volume of 255.95 ± 52.93 mm^3^ compared to the OE-NC group (434.34 ± 75.78 mm³), demonstrating the independent anti-tumor effect of MC1R knockdown. The NC + RMP1-14 group exhibited a tumor volume of 127.82 ± 42.50 mm^3^, establishing the baseline treatment response without ETV4 overexpression. These results confirmed that ETV4 overexpression significantly promotes tumor growth, while MC1R knockdown alone produces measurable growth inhibition.

To evaluate whether MC1R knockdown synergizes with immune checkpoint inhibitors in suppressing tumor growth, we compared treatment responses under ETV4-overexpressing conditions. ETV4-induced increases in tumor volume (416.54 ± 56.78 mm³) and weight (0.43 ± 0.05 g) were greatly attenuated by nivolumab (267.77 ± 40.99 mm³, 0.28 ± 0.06 g, *P* < 0.05). Similarly, the anti-PD-1 antibody RMP1-14 significantly suppressed ETV4-driven tumor growth, with trends consistent with the nivolumab group (*P* < 0.05). The combination therapy of OE-ETV4 + sh-MC1R + nivolumab/RMP1-14 resulted in the strongest suppression of tumor growth (118.86 ± 17.13 mm³, 0.13 ± 0.01 g, *P* < 0.001; 71.994 ± 16.793 mm^3^, 0.11 ± 0.02 g, *P* < 0.001, respectively), as shown in Figure 7 and Supplemental Digital Content Figure 5, available at: http://links.lww.com/JS9/G974. Furthermore, TAM depletion using the CSF1R inhibitor PLX3397 (OE-ETV4 + PLX3397 group) significantly inhibited tumor growth (166.913 ± 39.347 mm^3^, 0.22 ± 0.03 g, *P* < 0.01), as illustrated in Supplemental Digital Content Figures 5E-F, available at: http://links.lww.com/JS9/G974.

**Figure 8.**
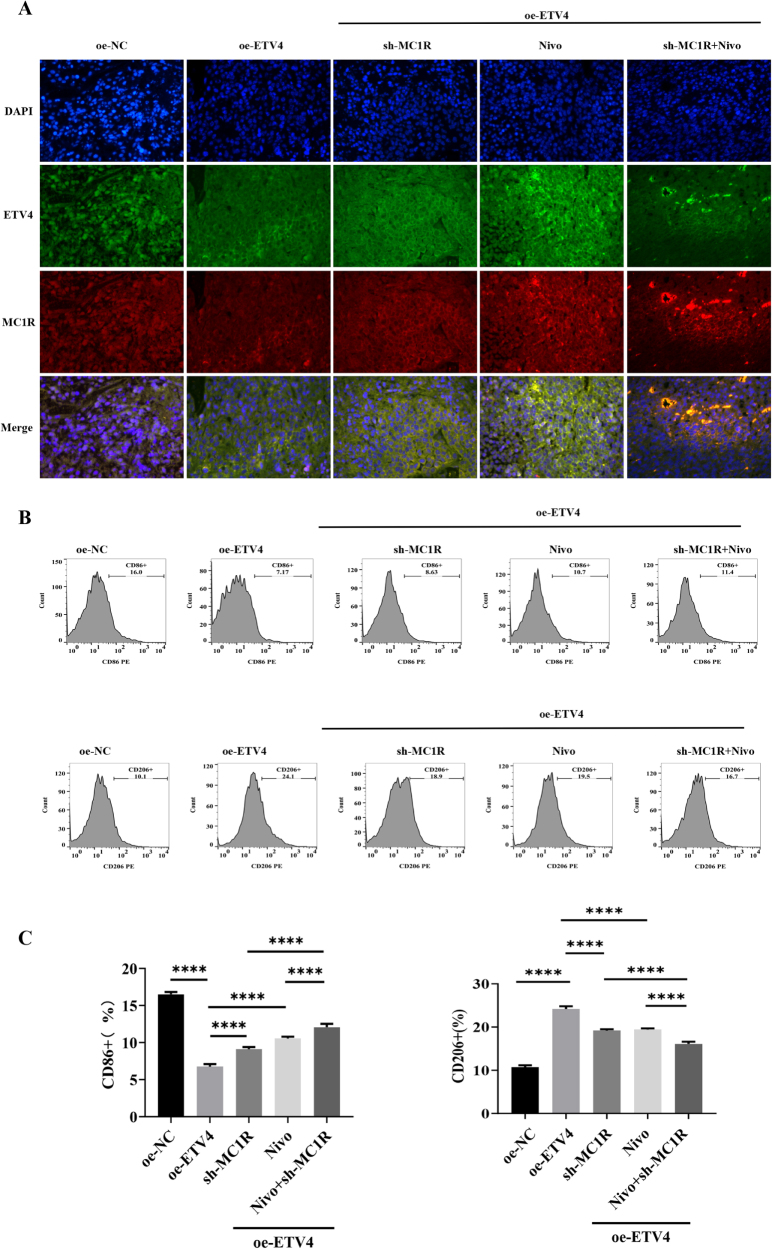
The ETV4–MC1R axis modulates macrophage polarization to promote resistance to PD-1 immunotherapy. (A) Immunofluorescence analysis of ETV4 and MC1R protein in tumor microenvironment (TME; *n* = 6). (B) Flow cytometry was performed to detect M1 (CD86⁺) macrophages and M2 (CD206⁺) macrophages (*n* = 6). (C) Quantitative statistical diagram for expression of M1 macrophages and M2 macrophages (*n* = 6).

qPCR and western blot analyses confirmed that ETV4 overexpression upregulated MC1R expression. In contrast, neither MC1R silencing nor PD-1 blocker treatment significantly altered ETV4 levels (Fig. 7G and Supplemental Digital Content Figure 5G, available at: http://links.lww.com/JS9/G974). Immunofluorescence assays yielded consistent results (Fig. 8A).

### The ETV4-MC1R axis modulates macrophage polarization to promote resistance to PD-1 therapy

To investigate whether ETV4-mediated tumor progression via MC1R involves macrophage reprogramming, we performed flow cytometry analysis of M1 (CD86⁺) and M2 (CD206⁺) macrophage populations. As shown in Figure 8B, ETV4 overexpression significantly reduced M1 macrophage infiltration in the TME (10.53 ± 0.37 vs. 2.48 ± 0.14, *P* < 0.0001) while enhancing M2 macrophage polarization (6 ± 0.17 vs. 13.42 ± 0.49, *P* < 0.01), indicating establishment of an immunosuppressive environment.

We next examined whether MC1R knockdown and PD-1 blocker could reverse ETV4-mediated macrophage polarization. Both MC1R knockdown and PD-1 blockade monotherapies significantly increased M1 macrophage differentiation (2.48 ± 0.14 vs. 5.09 ± 0.24; 2.48 ± 0.14 vs. 5.62 ± 0.26,*P* < 0.01) while suppressing M2 macrophage infiltration (13.42 ± 0.497 vs. 10.24 ± 0.43; 13.42 ± 0.49 vs. 10.62 ± 0.57, respectively; *P* < 0.01), as shown in Figure 8B and Supplemental Digital Content Figures 6A-B, available at: http://links.lww.com/JS9/G974. Notably, the combination of MC1R knockdown with PD-1 blockade demonstrated superior efficacy, significantly increasing M1 macrophages (5.62 ± 0.26% vs. 7.54 ± 0.44%, *P* < 0.001) and reducing M2 macrophage accumulation (10.62 ± 0.57% vs. 8.31 ± 0.35%, *P* < 0.05) compared to PD-1 blockade alone (Fig. 8B-C). Furthermore, TAM depletion using PLX3397 (OE-ETV4 + PLX3397 group) significantly reversed M2 macrophage polarization (13.47 ± 1.70 vs. 5.71 ± 0.31, *P* < 0.0001) and enhanced M1 macrophage infiltration (0.56 ± 0.06 vs. 1.38 ± 0.12, *P* < 0.0001), supporting the crucial role of TAMs in ETV4-MC1R-mediated immunosuppression (Supplemental Digital Content Figures 6C-D, available at: http://links.lww.com/JS9/G974).

qPCR analysis of tumor-associated macrophage (TAM)-secreted cytokines corroborated these findings. ETV4 overexpression elevated levels of the M2-associated cytokine IL-10 while suppressing M1-associated cytokines (IL-6, IL-1β, and TNF-α). Conversely, MC1R silencing promoted production of pro-inflammatory cytokines (IL-6, IL-1β, and TNF-α) while inhibiting IL-10 expression (Supplemental Digital Content Figures 4C-D, available at: http://links.lww.com/JS9/G974).

These results demonstrated that ETV4 promotes CRC progression by enhancing MC1R-dependent M2 macrophage polarization. Targeting the ETV4–MC1R axis synergizes with anti–PD-1 therapy to overcome immunosuppression, revealing a promising therapeutic strategy for CRC.

## Discussion

In this study, we employed scRNA-seq to delineate the transcriptional regulatory heterogeneity between PD-1-resistant and PD-1-sensitive CRC subpopulations. We identified markedly elevated transcriptional activity of specific regulatory genes, most notably ETV4, in epithelial cells from PD-1-resistant tumors. To translate these findings into clinical relevance, we integrated multiple machine learning algorithms to develop a prognostic model based on ETV4-associated transcriptional signatures, which demonstrated robust predictive performance across independent validation cohorts. The main strengths of this study include the integration of scRNA-seq with machine learning to develop a robust prognostic model, the identification of a novel ETV4-MC1R mechanism through direct promoter binding and macrophage reprogramming, and validation across independent cohorts using both transcriptomic and experimental approaches, providing a framework for personalized therapy in CRC.

It is important to note that this study primarily focuses on the mechanism of primary resistance, as all patient samples were derived from PD-1 inhibitor-naïve MSI-H metastatic CRC patients. The limited sample size precluded investigation of secondary resistance mechanisms and may affect the broader applicability of our results. Future prospective, multi-institutional studies with larger cohorts and standardized protocols are warranted to validate our prognostic model and systematically delineate the molecular networks underlying both primary and secondary resistance mechanisms.

Accumulating evidence has identified ETV4 (ETS variant TF 4) as a critical oncogenic driver across various malignancies, and its overexpression correlates with poor prognosis through the regulation of tumor proliferation, invasion, and therapy resistance^[26–28]^. In lung adenocarcinoma, ETV4 enhances PD-L1 expression via STAT3-mediated transcriptional activation^[29]^. ETV4 may modulate the expression of CXCL1 and CXCL8, facilitating the recruitment of immunosuppressive cells and fostering an immunosuppressive TME conducive to bladder cancer progression^[27]^. Through single-cell sequencing analysis combined with molecular experiments, we established that ETV4 overexpression creates an immunologically “cold” TME and enhances anti-PD-1 resistance by promoting MC1R expression and TAM infiltration^[30]^.

Although PD-1 inhibitors demonstrate durable therapeutic efficacy in MSI-H CRC, significant immunotherapy resistance persists in patients with MSS and partially advanced MSI-H CRC. This clinical challenge is strongly correlated with the characteristic immunosuppressive TME dominated by myeloid-derived suppressor cells (MDSCs) and TAMs^[12,31]^. This study revealed that activation of the ETV4-MC1R axis drives M2 macrophage polarization, emerging as a critical mechanism underlying the establishment of immunologically “cold” tumors and resistance to immune checkpoint inhibitors. Notably, M2-polarized macrophages secrete immunosuppressive cytokines such as IL-10, which directly inhibit cytotoxic T-cell activity while promoting the expansion of regulatory T-cells (Tregs), thereby dampening the efficacy of PD-1 blockade^[32–34]^. Conversely, M1-like macrophages produce proinflammatory cytokines, including TNF-α, IL-1β, and IL-6, which enhance antigen presentation and Th1-mediated anti-tumor immunity^[34–36]^. Our co-culture experiments demonstrated that ETV4-overexpressing CRC cells skewed macrophage polarization toward an IL-10-high/M2 phenotype while suppressing the secretion of TNF-α, IL-1β, and IL-6, establishing a direct link between ETV4-MC1R signaling and cytokine-mediated immunosuppression. Compared with PD-1 inhibitor monotherapy, combination therapy targeting both the PD-1 and ETV4-MC1R axes resulted in superior tumor growth suppression, suggesting that dual-pathway blockade is a viable strategy for overcoming therapeutic resistance. These findings have significant clinical implications, as targeting the ETV4-MC1R axis could lead to new combination therapies to overcome PD-1 resistance.

While our study convincingly demonstrates that the ETV4-MC1R axis promotes M2 macrophage polarization, we recognize that other immune cell subsets, such as regulatory T cells (Tregs) and MDSCs, may also contribute to immunotherapy resistance. Although our focus was on the role of ETV4-driven MC1R activation in M2 polarization, the immunosuppressive TME involves multiple factors. For example, Tregs are known to suppress cytotoxic T-cell activity and promote immune tolerance, while MDSCs can inhibit T-cell function and enhance Treg expansion**^[37,38]^**. This multifactorial nature of resistance suggests that combinatorial approaches targeting additional immune populations could enhance therapeutic outcomes. Beyond the ETV4-MC1R axis, emerging evidence suggests that multiple mechanisms contribute to immunotherapy resistance. Metabolic pathways such as glycolysis and glutamine metabolism have been implicated in therapy resistance^[39]^, and gut microbiota modulation has emerged as a strategy to improve immune checkpoint blockade outcomes^[40]^. These findings highlight the multifactorial nature of treatment resistance and suggest potential combinatorial approaches targeting both tumor-intrinsic pathways and microenvironmental factors.

Melanocortin 1 receptor (MC1R), a G protein-coupled receptor, has emerged as a critical regulatory component in multiple cancer types^[41,42]^. The activation of MC1R signaling triggers the secretion of immunosuppressive cytokines, including IL-10 and TGF-β, from melanoma cells, effectively suppressing macrophage M1 polarization^[43–45]^. Notably, preclinical studies have demonstrated enhanced tumor growth suppression through the combined inhibition of MC1R and anti-PD-1 antibody therapy in murine models^[46]^. Mechanistically, we identified ETV4 as a direct transcriptional activator of MC1R through promoter binding, thereby establishing a novel pathway that facilitates tumor progression. While further preclinical and clinical evaluation is required, these findings underscore the therapeutic potential of dual-targeting ETV4 or MC1R in conjunction with immunotherapeutic strategies.

Although our study integrated single-cell sequencing and transcriptomic profiling with experimental verification, several limitations warrant consideration. The comparative analysis of PD-1 resistance mechanisms was limited by the modest sample size of clinical specimens. Further validation through larger, multi-center cohorts across diverse treatment stages is needed to confirm generalizability and assess the clinical translatability of dual-targeting strategies. Although our transcriptomics-based prognostic model demonstrated robust performance in public cohorts, its clinical utility requires confirmation through prospective, multi-institutional validation studies with standardized therapeutic protocols. Furthermore, MC1R expression in normal tissues (particularly skin) raises potential safety concerns for targeted therapies, warranting comprehensive preclinical toxicity evaluation. Additionally, the lack of spatial transcriptomic data limited our ability to resolve the spatial relationships between ETV4^+^ cells and macrophages in the TME. Future studies should employ spatial transcriptomics to validate cellular interactions and systematically assess the potential toxicity of MC1R-targeted therapies.

Overall, our study not only reconstructs the regulatory circuitry of PD-1 resistance at single-cell resolution but also highlights the need for larger-scale validation to establish clinical relevance.

## Conclusion

By integrating single-cell transcriptomes from PD-1-treated MSI-H metastatic CRCs, we systematically reconstructed GRNs and revealed a resistance-specific program driven by the TF ETV4. ETV4 was preferentially active in malignant epithelial cells and correlated strongly with macrophage-associated signatures across TCGA cohorts. Functional studies have demonstrated that ETV4 skews macrophages toward an immunosuppressive M2 phenotype and promotes immune evasion by directly upregulating MC1R. Disruption of the ETV4-MC1R axis, either genetically or in combination with anti-PD-1 therapy, restores antitumor immunity and suppresses tumor growth *in vitro* and *in vivo*. Collectively, our work identified the ETV4-MC1R pathway as a critical driver of PD-1 resistance in CRC, highlighting it as a tractable target for next-generation immunotherapeutic strategies.

## Data Availability

The data availability statement has been supplemented at the corresponding position in the original text.
